# Socioeconomic status and motivation in endurance sports: insights from long-distance running

**DOI:** 10.3389/fpsyg.2025.1527661

**Published:** 2025-03-26

**Authors:** Anna Akbaş, Aleksandra Żebrowska, Ewa Malchrowicz-Mośko, Jakub Stempień, Eduard Bezuglov, Agnieszka Górka-Chowaniec, Zbigniew Waśkiewicz

**Affiliations:** ^1^Institute of Sport Science, Jerzy Kukuczka Academy of Physical Education, Katowice, Poland; ^2^Department of Tourism and Recreation, Faculty of Physical Education, Józef Piłsudski University of Physical Education, Warsaw, Poland; ^3^The Department of Rural and Urban Sociology, Institute of Sociology, Faculty of Economics and Sociology, University of Lodz, Łódź, Poland; ^4^Department of Sports Medicine and Medical Rehabilitation, Sechenov First Moscow State Medical University, Moscow, Russia; ^5^Sport and Tourism Management Faculty, Jerzy Kukuczka Academy of Physical Education, Katowice, Poland; ^6^Department of Management Theory, Institute of Sport Science, Jerzy Kukuczka Academy of Physical Education, Katowice, Poland

**Keywords:** marathon, running, motivational factors, socioeconomic status, endurance sports

## Abstract

**Introduction:**

Endurance running is a popular activity with varying motivations, yet the influence of socioeconomic status (SES) on these motivations has not been thoroughly explored. This study investigates how SES (income, education, vocational status) shapes the motivations of three groups of runners: recreational runners (RRs), marathoners (MAs), and ultramarathoners (Us) in Poland.

**Methods:**

A large-scale online survey (*N* = 1,539) was conducted between January and March 2008, capturing participants’ demographic characteristics, running experience, and motivation.

**Results:**

The results reveal differences in motivational priorities across groups. Us were more motivated by achievement and self-esteem, while RRs prioritized social affiliation, and marathoners showed a balance between the two. However, the reported effect sizes (*η*^2^ = 0.01) suggest that while differences exist, they are small in practical significance. Higher-SES individuals focused on achievement-related goals, while lower-SES individuals emphasized health benefits and social connection. The analysis also found that higher-SES participants were more likely to complete marathons and ultramarathons, with financial stability and professional access playing a key role.

**Discussion:**

Nevertheless, ultramarathon participation appeared to depend more on intrinsic motivation than financial resources. These findings underline the complex interplay between SES, personality, and experience, emphasizing the need for tailored strategies to support diverse runners. Further research may explore the broader psychological and cultural factors influencing running motivations.

## Introduction

The global surge in recreational running and competitive events, such as marathons and ultramarathons, has become a defining feature of modern sport, reflecting the pervasive ideology of health and fitness as central to personal responsibility and moral virtue (Crawford, 1980; McRae and Gross, 2020). While the motivations behind running have been extensively studied, particularly among elite athletes (Braschler et al., 2024; Partyka and Waśkiewicz, 2024), less attention has been given to the role of socio-economic status (SES) in shaping the motivations of recreational runners (RRs), marathoners (MAs), and ultramarathoners (Us). SES, encompassing factors like income, education, and material wellbeing, can influence accessibility to endurance sports, participation in competitive events, and the prioritization of intrinsic motivators such as personal goal achievement or self-esteem (Krouse et al., 2011; Roebuck et al., 2018). Understanding how SES impacts these motivations is essential for fostering inclusive and supportive frameworks encouraging broader participation in endurance sports (Eagly and Wood, 2012; Osbaldiston and Sheldon, 2003; Simpkins et al., 2010; Ullrich et al., 2022). SES is a well-documented determinant of motivation across multiple domains, particularly in educational psychology. Research indicates that individuals from higher-SES backgrounds often exhibit higher levels of intrinsic motivation due to greater access to resources, parental support, and enriched learning environments (Deci and Ryan, 1985; Eccles and Wigfield, 2002). In the domain of learning, SES influences children’s motivation through parental expectations and involvement (Simpkins et al., 2010). Families with higher SES are more likely to emphasize goal-setting, discipline, and long-term achievement, all of which align with motivational structures observed in endurance athletes (Duncan et al., 1994). This parental influence can translate into sports participation, where high-SES individuals may develop stronger goal-oriented motivation patterns (Fredricks and Eccles, 2005).

Furthermore, the self-determination theory (Deci and Ryan, 1985) suggests that intrinsic motivation is fostered when individuals experience autonomy, competence, and relatedness. High-SES individuals often have more opportunities to engage in activities that promote these psychological needs, such as structured athletic training programs and professional coaching, enhancing motivation for endurance sports (Hagger and Chatzisarantis, 2007). Pierre Bourdieu’s concept of cultural capital provides another lens for understanding how SES influences motivation in sports (Bourdieu, 1984). Higher-SES individuals accumulate cultural capital through early exposure to organized sports, access to elite training facilities, and social networks that encourage participation in sports (Coakley, 2011). These advantages foster a long-term commitment to endurance sports, often driven by achievement motivation rather than mere recreational interest (Stempel, 2005). Moreover, financial capital plays a critical role in sports engagement. Participation in endurance sports such as marathons and ultramarathons often requires substantial financial investment, including entry fees, travel costs, equipment, and specialized training (Breuer et al., 2010). As a result, individuals from higher-SES backgrounds are more likely to engage in these activities because they can afford them and perceive them as valuable forms of self-improvement and social distinction (Wilson, 2002).

Motivation in the running includes several key dimensions, ranging from health-related goals, such as physical and psychological wellbeing, to social and achievement-oriented motivations (Jastifer and Hoffman, 2025). For many recreational runners, the pursuit of health is a primary driver (Dobrosz, 2019; Stempień et al., 2020), while others may be motivated by the sense of community that running events provide (Malchrowicz-Mośko and Waśkiewicz, 2020; Zduniak, 2010). In more competitive contexts, such as marathons and ultramarathons, motivations related to self-improvement, personal achievement, and self-esteem become more pronounced (Waśkiewicz et al., 2018; Gerasimuk et al., 2021). However, SES appears to play a significant role in how these motivations are prioritized. Higher-SES individuals are more likely to focus on achievement and self-worth, as they often have greater access to resources such as coaching, race fees, and travel opportunities (Koech et al., 2024; Maklári et al., 2024). In contrast, lower-SES participants emphasize health, stress relief, or social connections more, driven by the fewer resources available for competitive engagement (Simpkins et al., 2010; Smith et al., 2009). SES also interacts with psychological wellbeing, which influences long-term motivation for sports participation. Research has shown that high-SES individuals have lower stress levels and greater access to psychological coping resources (Jiang et al., 2020). These factors contribute to sustained engagement in endurance sports, where persistence and self-regulation are essential for success (Brick et al., 2014). Conversely, lower-SES individuals may experience motivational barriers due to financial stress, limited access to training facilities, and competing life demands (Eime et al., 2010). However, for some, endurance sports serve as a coping mechanism for socioeconomic adversity, providing an outlet for stress relief and social connection (Cope et al., 2017). This suggests that while higher-SES individuals may be more motivated by goal achievement and self-esteem, lower-SES individuals may prioritize health benefits and community engagement.

Despite the recognized impact of SES on various aspects of participation in endurance sports, its influence on the motivations of RRs, MAs, and Us remains underexplored. Given the increasing popularity of running and the diverse demographic groups it attracts, assessing how variations in SES (higher or lower levels of economic, vocational, and educational indicators) affect how people engage with and prioritize different motivational factors is essential. Therefore, this study aims to fill this gap by exploring how SES influences the motivations of RRs, MAs, and Us. We will examine (1) the SES characteristics of RRs, Mas, Us, (2) the key motivational factors driving each group, (3) how socioeconomic status shapes the importance of these motivations both in general and within each group, and (4) the relationship between SES and the number of completed marathons and ultramarathons. We hypothesize that (1) socioeconomic characteristics will significantly differ across RRs, MAs, and Us, with higher SES individuals more likely to participate in more demanding endurance sports; (2) marathon and ultramarathon runners will exhibit higher levels of goal achievement, affiliation, life meaning, and self-esteem motivations than recreational runners due to the goal-oriented nature of these endurance events. We further hypothesize that (3) SES will significantly influence motivational factors in endurance sports, with higher-SES individuals prioritizing achievement and self-esteem and lower-SES individuals focusing on health improvement and social affiliation due to resource limitations and community engagement. Finally, we hypothesize that (4) higher SES will be positively associated with the number of completed marathons and ultramarathons, reflecting greater access to resources facilitating participation in these events.

## Materials and methods

### Study design and sample

This cross-sectional online survey followed the CHERRIES guidelines (Checklist for Reporting Results of Internet E-Surveys) (Eysenbach, 2004). Participants were eligible if they were at least 18 years old, residing in Poland, and had been engaged in running at varying levels for at least 3 months. Exclusion criteria included incomplete survey responses. The study utilized a convenience sampling method, with recruitment through online advertisements and marathon event organizers. As the survey was openly accessible, the number of invited participants could not be determined. Ultimately. The study included data from a total of 1,539 runners.

Participants were grouped based on their endurance running engagement: RRs, MAs, and Us. RRs were defined as individuals engaged in running for leisure or fitness without completing a marathon, MAs as those who had completed at least three marathons (42.195 km), and Us as those who had completed at least three races beyond the marathon distance. While these categories are generally distinct, there may be overlap between MAs and Us; in such cases, participants were classified as ultramarathoners, reflecting their highest level of engagement. The distinction between recreational runners, marathoners, and ultramarathoners is rooted in previous research on endurance sports (Krouse et al., 2011; Gerasimuk et al., 2021). Recreational runners engage in running primarily for health and social reasons, while marathoners typically demonstrate goal-driven behaviors with structured training regimens. Ultramarathoners, in contrast, are often characterized by extreme intrinsic motivation, perseverance, and a strong sense of self-achievement (Zach et al., 2017). By including all three categories, we aim to examine how SES influences motivational factors across different levels of endurance sports commitment. The demographic characteristics of the sample are provided in Table 1.

**Table 1:** Demographic characteristics of recreational runners (RRs), marathoners (MAs), and ultramarathoners (Us).

Characteristics	Groups		
	RRs	MAs	Us
	(*n* = 277)	(*n* = 839)	(*n* = 423)
Sex, *n* (%)			
Female	98 (35.38%)	200 (23.84%)	83 (19.62%)
Male	179 (64.62%)	637 (76.16%)	340 (80.83%)
Age, *n* (%)			
Less than 30	120 (43.32%)	185 (22.05%)	61 (14.42%)
31–40 years	90 (32.49%)	315 (37.54%)	146 (34.52%)
41–50 years	56 (20.22%)	254 (30.27%)	160 (37.83%)
More than 51 years	11 (3.97%)	85 (10.13%)	56 (13.24%)

### Ethical aspects

This study was approved by the Bioethical Committee at the Academy of Physical Education in Katowice (KB/47/17), and all procedures were performed in accordance with the Declaration of Helsinki. The study adhered to ethical standards for online data collection, ensuring that participants were fully informed about the study’s aims, the confidentiality and security of their responses, their voluntary participation, and their right to withdraw at any time. These details were outlined in the Participant Information Statement provided before survey completion. By completing the survey, participants gave their informed consent to participate.

### Data collection

The survey was distributed through professional running websites and marathon organizers from January to March 2008. The inclusion of participants in several Polish marathons (for example, in Cracow, Wrocław, Poznań, and Silesia) was achieved through the utilization of various running-related websites, including ultraroztocze.pl, biegrzeznika.pl, maratonypolskie.pl, began.pl, biegologia.pl, polskabiega.pl, treningbiegacza.pl, wszystkoobieganiu.pl, biegaczki.pl, ultrabieganie.pl, and festiwalbiegowy.pl. The survey was open to all runners residing in Poland with internet access. Information about the significance of the study was provided to the participants, who were kindly requested to divulge information regarding their sex, age, education, training experience, training frequency, and income. The survey was open to all runners residing in Poland with internet access.

The MOMS questionnaire contained 56 items distributed across nine scales (Masters et al., 1993). These nine motivations were divided into four main categories: (1) physical motives for running, which include general health benefits and weight concerns; (2) achievement-related motives, which include competition with other runners and achievement of personal goals; (3) social motives, which include the desire to affiliate with other runners and receive recognition or approval from others; and (4) psychological motives, which include maintaining or enhancing self-esteem, providing a sense of life meaning, and problem-solving or coping with negative emotions. The MOMS was translated into Polish, its reliability was verified, and the participants were asked to answer it (Dybała, 2013) using a seven-point Likert-type scale from 1 (“not a reason”) to 7 (“most important reason”). We measured the reliability of the collected data using Cronbach’s alpha coefficients of internal consistency, showing “good” (0.8–0.9) and “excellent” levels (above 0.9) depending on the category. A pilot study was not necessary, as the Polish version of the MOMS questionnaire had already been validated (Dybała, 2013) and has been successfully used in previous research (Gerasimuk et al., 2021; Waśkiewicz et al., 2018, 2019).

Prior to completing the MOMS questionnaire, we evaluated their socioeconomic status based on their educational level, type of professional activity, and net household income per capita. The participants graded their material wellbeing on a five-point scale and expressed their attitudes toward material needs. Education level pertained to whether the participants had achieved higher or secondary education. Regarding professional activity, the respondents were grouped into those with full-time employment (i.e., working a standard number of hours per week, commonly 35–40), self-employment (i.e., operating their own business or working as independent contractors, freelancers, or sole proprietors), students, or unemployed (i.e., not engaged in any gainful employment or occupation that provides regular income or retired). Concerning material status and needs, the respondents reported their incomes as higher or lower than 3,000 PLN per capita per month [as the average income stated by Statistics Poland (Statistics Poland, 2018)], graded their material wellbeing on a five-point scale (Table 1), and expressed their importance of material needs (important vs. not important to me).

### Statistical procedures

All data were analyzed using Statistica v.13.3 (TIBCO Software Inc.). Group homogeneity in socioeconomic characteristics was assessed using chi-square tests for nominal data. The same test was applied to evaluate differences in the number of completed marathons and ultramarathons across socioeconomic characteristics. Post-hoc pairwise chi-square tests were conducted where necessary, and Bonferroni correction was applied to control for multiple comparisons. Effect sizes for the chi-square tests were reported as Cohen’s *ω*, with values interpreted as follows: 0.1 (small), 0.3 (medium), and 0.5 (large).

Differences in motivation types among the RRs, MAs, and Us groups and across socioeconomic characteristics were analyzed using the Kruskal–Wallis mean rank test with Dunn’s post-hoc comparisons. Effect sizes were reported as eta squared (*η*^2^), with values interpreted as follows: 0.01 to <0.06 (small effect), 0.06 to <0.14 (moderate effect), and ≥0.14 (large effect).

Spearman’s rank correlation coefficient was used to explore the relationship between socioeconomic status and motivation type within the RR, MA, and U groups.

A *post hoc* power analysis was conducted using G*Power 3.1.9.7 (Heinrich-Heine-Universität Düsseldorf, Germany) to assess the sensitivity of chi-square tests and Kruskal-Wallis tests, based on the observed sample sizes and a small effect size. The analysis showed that all chi-square tests had a post-hoc power of 0.8 or greater, and all Kruskal-Wallis tests had a post-hoc power greater than 0.9, indicating that the study was adequately powered to detect significant effects (Cohen, 2013; Faul et al., 2007). A significance level of *p* < 0.05 was adopted for all statistical tests.

## Results

The results of this study present a comprehensive analysis of the relationship between SES and motivational factors among various types of runners (RRs, MAs, Us). The results are organized into several key subsections. First, we explore the differences in SES across the three groups of runners. Next, we examine the overall differences in motivational factors among the groups. Following this, we investigate how SES characteristics relate to motivational differences both in general and within each group. We then analyze the relationship between SES and motivation in greater detail for each type of runner. Finally, we explore the differences in the number of completed marathons and ultramarathons across SES characteristics.

### Group differences in socioeconomic characteristics

We compared the RRs, MAs, and Us groups regarding socioeconomic status, including education, professional activity, net income per capita, material wellbeing, and attitudes toward material needs.

The only significant difference in the distribution among the three groups was observed in the type of professional activity (*p* < 0.001) (Table 2). Full-time employed individuals accounted for the highest percentage of all three groups. The second most common activity was self-employment in the MAs and Us groups, whereas students formed the second largest percentage in the RRs group. Unemployed individuals had the smallest percentage in the RR and MAs groups but the second smallest percentage in the U group. All groups showed no differences in education, net income per capita, material wellbeing, or attitude toward material needs (*p* > 0.05) (Table 2).

**Table 2:** Socioeconomic characteristics of recreational runners (RRs), marathoners (MAs), and ultramarathoners (Us).

Variables	Groups			*p* (*ω*)
	RRs	MAs	Us	
	(*n* = 277)	(*n* = 839)	(*n* = 423)	
Education, *n* (%)				0.308 (0.04)
Higher	192 (69.31%)	614 (73.18%)	315 (74.47%)	
Secondary	85 (30.69%)	225 (26.82%)	108 (25.53%)	
Professional activity, *n* (%)				<0.001 (0.18)
Full-time employment	201 (72.56%)	659 (78.55%)	326 (77.07%)	
Self-employment	19 (6.86%)	97 (11.56%)	58 (13.71%)	
Student	50 (18.05%)	61 (7.27%)	19 (4.49%)	
Unemployed	7 (2.53%)	22 (2.62%)	20 (4.73%)	
Net income per capita, *n* (%)				0.052 (0.06)
>3 k	82 (29.60%)	315 (37.54%)	156 (36.88%)	
≤3 k	195 (70.40%)	524 (62.46%)	267 (63.12%)	
Material wellbeing, *n* (%)				0.277 (0.07)
I can afford everything I dream of	6 (2.17%)	34 (4.05%)	13 (3.07%)	
I satisfy my needs to a satisfactory degree	41 (14.80%)	104 (12.40%)	53 (12.53%)	
I satisfy my needs to the minimum extent	208 (75.09%)	645 (76.88%)	338 (79.91%)	
I am dealing, but I often have financial problems	22 (7.94%)	56 (6.67%)	19 (4.49%)	
Attitude toward material needs, *n* (%)				0.13 (0.05)
Important to me	137 (49.46%)	417 (49.70%)	186 (43.97%)	
Irrelevant to me	140 (50.54%)	422 (50.30%)	237 (56.03%)	

RR, non-starters; MA, marathoners; U, ultramarathoners; net income per capita reported in Polish złoty (PLN); *ω*, effect size; significant results are shown in bold (*p* < 0.05).

### Group differences in motivation

This analysis aimed to determine which motivational drivers—physical, achievement-oriented, social, or psychological—were most prominent in each group of runners (RRs, MAs, Us) and whether distinct patterns emerged based on race type.

Most motivation scores across the groups exceeded 3.5, positioning them in the upper range of the 7-point scale. Notably, the mean scores for the sense of competition and social motivations associated with recognition and affiliation were approximately 3.5 or lower.

The dominant motivation observed in each group was personal goal achievement, which had the highest mean and median values. Interestingly, the groups significantly differed in the extent of personal goal achievement, with the Us group showing substantially higher motivation due to personal goal achievement than the MAs and RRs groups (*p* < 0.001). No significant differences were found between the MAs and RRs groups regarding personal goal achievement (*p* > 0.05). A similar observation was made for self-esteem, with the Us group significantly differing from both the MAs and RRs groups (*p* < 0.05) and the MAs and RRs groups showing no significant differences (*p* > 0.05). Although these differences were statistically significant, all effect sizes were small (exact values are provided in Table 3).

**Table 3:** Differences in Motivations of Marathoners Scales (MOMS) between recreational runners (RRs), marathoners (MAs), and ultramarathoners (Us) based on the Kruskal-Wallis test.

Categories	Groups							
	RRs	MAs	Us	Main effect	Effect size	RR vs MA	RR vs U	MA vs U
	mean ± SD	mean ± SD	mean ± SD	H	*η* ^2^	*p*	*p*	*p*
	(mdn, min, max)	(mdn, min, max)	(mdn, min, max)	*p*				
Physical								
Health orientation	4.55 ± 1.07	4.67 ± 1.06	4.67 ± 1.09	2.95	0.00	—	—	—
	(4.83, 1.5, 7.0)	(4.83, 1.0, 7.0)	(4.83, 1.0, 7.0)	*0.23*				
Weight concern	4.32 ± 1.66	4.68 ± 1.62	4.51 ± 1.63	14.68	0.01	>0.05	> 0.05	**<0.001**
	(4.25, 1.0, 7.0)	(5.00, 1.0, 7.0)	(4.75, 1.0, 7.0)	**<0.001**				
Achievement								
Personal goal achievement	5.08 ± 1.25	5.40 ± 1.16	5.46 ± 1.17	24.87	0.01	>0.05	**<0.001**	**<0.001**
	(5.17, 1.2, 7.0)	(5.67, 1.0, 7.0)	(5.67, 1.0, 7.0)	**<0.001**				
Competition	3.35 ± 1.63	3.37 ± 1.65	3.31 ± 1.70	0.46	0.00	—	—	—
	(3.25, 1.0, 7.0)	(3.25, 1.0, 7.0)	(3.00, 1.0, 7.0)	*0.79*				
Social								
Recognition	2.94 ± 1.25	3.04 ± 1.29	3.08 ± 1.37	1.85	0.00	—	—	—
	(2.67, 1.0, 6.8)	(2.83, 1.0, 6.8)	(2.83, 1.0, 6.7)	*0.4*				
Affiliation	3.55 ± 1.60	3.38 ± 1.62	3.23 ± 1.62	6.57	0.00	>0.05	**0.036**	> 0.05
	(3.50, 1.0, 7.0)	(3.33, 1.0, 7.0)	(3.00, 1.0, 7.0)	**0.037**				
Psychological								
Psychological coping	4.26 ± 1.46	4.43 ± 1.46	4.35 ± 1.59	3.67	0.00	—	—	—
	(4.33, 1.0, 7.0)	(4.56, 1.0, 7.0)	(4.44, 1.0, 7.0)	*0.16*				
Life meaning	4.19 ± 1.39	4.04 ± 1.43	3.99 ± 1.49	4.64	0.00	—	—	—
	(4.29, 1.0, 7.0)	(4.14, 1.0, 7.0)	(4.00, 1.0, 7.0)	*0.098*				
Self esteem	4.43 ± 1.36	4.67 ± 1.36	4.69 ± 1.44	9.97	0.01	>0.05	**0.043**	**0.009**
	(4.50, 1.0, 7.0)	(4.88, 1.0, 7.0)	(4.75, 1.0, 7.0)	**0.007**				

RR, recreational runners; MA, marathon runners; U, ultramarathon runners; SD, standard deviation; mdn, median; min, minimum; max, maximum; H, Kruskal-Wallis statistics; significant results are shown in bold (*p* < 0.05).

Weight concerns were a motivating factor that distinguished the Us group from the RRs group (*p* < 0.001). However, the MAs group did not differ from the other two groups (*p* > 0.05). Finally, while affiliation was far from dominant motivation (with mean scores of around 3.5) in all groups, there was a significant difference between the RRs and Us groups (*p* < 0.05). Similarly, the effect sizes for these differences were also small (see Table 3).

The scores for other motivations, such as health orientation, competition, recognition, psychological coping, and life meaning, did not significantly vary between the groups (*p* > 0.05). Table 3 summarizes the results.

### General differences in motivations across different socioeconomic characteristics

The analysis revealed significant associations between socioeconomic characteristics and motivational dimensions measured by the MOMS for all runners together. Education was a particularly influential factor, with participants holding higher education levels scoring significantly higher in affiliation, life meaning, and self-esteem (*p* < 0.05). These results suggest that education contributes to a broader sense of purpose and interpersonal connection in endurance sports. Furthermore, students displayed significantly higher scores in personal goal achievement compared to full-time employees, self-employed individuals, and unemployed participants (*p* < 0.05). This indicates that students may be uniquely driven by personal development and achievement goals (Table 4).

**Table 4:** Differences in Motivations of Marathoners Scales (MOMS) across socioeconomic characteristics.

Main effects	Categories								
	HO	WC	PGA	COM	REC	AFF	PC	LM	SE
Education	*U* = 227,722 *p* = 0.397 *η*^2^ < 0.01	*U* = 233,345.5 *p* = 0.903 *η*^2^ < 0.01	*U* = 223,961 *p* = 0.183 *η*^2^ < 0.01	*U* = 226,222 *p* = 0.298 *η*^2^ < 0.01	*U* = 221,170 *p* = 0.091 *η*^2^ < 0.01	*U* = 206,194.5 ***p* < 0.001** *η*^2^ = 0.01	*U* = 232,106.5 *p* = 0.778 *η*^2^ < 0.01	*U* = 209,490.5 ***p* = 0.001** *η*^2^ = 0.01	*U* = 215,784 ***p* = 0.017** *η*^2^ < 0.01
Higher (mean ± SD)	4.62 ± 1.07	4.55 ± 1.62	5.31 ± 1.17	3.32 ± 1.61	2.99 ± 1.28	3.3 ± 1.58	4.36 ± 1.45	4 ± 1.39	4.56 ± 1.36
Secondary (mean ± SD)	4.68 ± 1.07	4.55 ± 1.7	5.37 ± 1.24	3.46 ± 1.77	3.12 ± 1.32	3.66 ± 1.7	4.38 ± 1.58	4.27 ± 1.52	4.75 ± 1.41
Professional activity	*H* = 5.62 *p* = 0.132 *η*^2^ < 0.01	*H* = 1.3 *p* = 0.728 *η*^2^ < 0.01	*H* = 20.44 ***p* < 0.001** *η*^2^ = 0.01	*H* = 1.96 *p* = 0.581 *η*^2^ < 0.01	*H* = 5.43 *p* = 0.143 *η*^2^ < 0.01	*H* = 4.73 *p* = 0.193 *η*^2^ < 0.01	*H* = 10.15 ***p* = 0.017** *η*^2^ < 0.01	*H* = 9.94 ***p* = 0.019** *η*^2^ < 0.01	*H* = 16.57 ***p* = 0.001** *η*^2^ = 0.01
Full-time employment (mean ± SD)	4.63 ± 1.07	4.54 ± 1.64	5.31 ± 1.18	3.33 ± 1.62	2.98 ± 1.27	3.4 ± 1.6	4.34 ± 1.47	4.05 ± 1.42	4.58 ± 1.35
Self-employment (mean ± SD)	4.75 ± 1.08	4.63 ± 1.67	5.24 ± 1.27	3.33 ± 1.7	3.16 ± 1.35	3.45 ± 1.72	4.32 ± 1.56	3.98 ± 1.48	4.49 ± 1.54
Student (mean ± SD)	4.53 ± 1.04	4.59 ± 1.67	5.72 ± 0.93	3.58 ± 1.79	3.21 ± 1.36	3.17 ± 1.51	4.75 ± 1.51	4.45 ± 1.41	5.1 ± 1.21
Unemployed (mean ± SD)	4.76 ± 1.07	4.38 ± 1.59	4.77 ± 1.54	3.32 ± 1.88	3.04 ± 1.36	3.76 ± 1.85	4.19 ± 1.47	4.04 ± 1.5	4.48 ± 1.57
Net income per capita	*U* = 268,191 *p* = 0.596 *η*^2^ < 0.01	*U* = 239,988.5 ***p* < 0.001** *η*^2^ = 0.01	*U* = 263,656 *p* = 0.283 *η*^2^ < 0.01	*U* = 271,234.5 *p* = 0.868 *η*^2^ < 0.01	*U* = 270,865 *p* = 0.833 *η*^2^ < 0.01	*U* = 239,305 ***p* < 0.001** *η*^2^ = 0.01	*U* = 268,631 *p* = 0.633 *η*^2^ < 0.01	*U* = 256,181.5 ***p* = 0.0493** *η*^2^ < 0.01	*U* = 260,459.5 *p* = 0.146 *η*^2^ < 0.01
>3 k (mean ± SD)	4.65 ± 1.09	4.76 ± 1.64	5.37 ± 1.18	3.34 ± 1.64	3.01 ± 1.3	3.18 ± 1.61	4.34 ± 1.48	3.97 ± 1.4	4.53 ± 1.38
≤3 k (mean ± SD)	4.63 ± 1.06	4.44 ± 1.63	5.3 ± 1.2	3.36 ± 1.66	3.03 ± 1.29	3.52 ± 1.61	4.38 ± 1.49	4.13 ± 1.45	4.66 ± 1.37
Material wellbeing	*H* = 8.44 *p* = 0.038 *η*^2^ < 0.01	*H* = 7.02 *p* = 0.071 *η*^2^ < 0.01	*H* = 10.15 ***p* = 0.017** *η*^2^ < 0.01	*H* = 8.87 ***p* = 0.031** *η*^2^ < 0.01	*H* = 0.65 *p* = 0.886 *η*^2^ < 0.01	*H* = 2.6 *p* = 0.458 *η*^2^ < 0.01	*H* = 7.04 *p* = 0.071 *η*^2^ < 0.01	*H* = 3.06 *p* = 0.383 *η*^2^ < 0.01	*H* = 6.6 *p* = 0.086 *η*^2^ < 0.01
I can afford everything I dream of	4.9 ± 1.23	4.97 ± 1.79	5.2 ± 1.29	3.38 ± 1.82	3.09 ± 1.38	3.61 ± 1.81	4.2 ± 1.53	3.92 ± 1.46	4.43 ± 1.54
I satisfy my needs to a satisfactory degree	4.62 ± 1.05	4.57 ± 1.62	5.36 ± 1.17	3.4 ± 1.64	3.01 ± 1.27	3.36 ± 1.59	4.33 ± 1.49	4.06 ± 1.41	4.58 ± 1.35
I satisfy my needs to the minimum extent	4.79 ± 1.09	4.27 ± 1.85	5.38 ± 1.32	3.5 ± 1.96	3.16 ± 1.47	3.61 ± 1.81	4.68 ± 1.59	4.27 ± 1.71	4.89 ± 1.6
I am dealing, but I often have financial problems (mean ± SD)	4.56 ± 1.15	4.5 ± 1.63	5.12 ± 1.2	3.01 ± 1.48	3.03 ± 1.33	3.47 ± 1.63	4.5 ± 1.4	4.11 ± 1.4	4.71 ± 1.35
Attitude toward material needs	*U* = 284,074.5 *p* = 0.185 *η*^2^ < 0.01	*U* = 244,987 ***p* < 0.001** *η*^2^ = 0.02	*U* = 262,737 ***p* < 0.001** *η*^2^ = 0.01	*U* = 246,871.5 ***p* < 0.001** *η*^2^ = 0.02	*U* = 256,856 ***p* < 0.001** *η*^2^ = 0.01	*U* = 288,138 *p* = 0.389 *η*^2^ < 0.01	*U* = 292,692.5 *p* = 0.736 *η*^2^ < 0.01	*U* = 291,998 *p* = 0.677 *η*^2^ < 0.01	*U* = 271,045 ***p* = 0.005** *η*^2^ = 0.01
Important to me (mean ± SD)	4.6 ± 1.07	4.33 ± 1.64	5.2 ± 1.25	3.13 ± 1.61	2.87 ± 1.22	3.44 ± 1.65	4.35 ± 1.49	4.05 ± 1.48	4.51 ± 1.41
Irrelevant to me (mean ± SD)	4.67 ± 1.07	4.8 ± 1.61	5.45 ± 1.11	3.6 ± 1.67	3.19 ± 1.35	3.36 ± 1.59	4.39 ± 1.48	4.1 ± 1.38	4.72 ± 1.33

HO, Health Orientation; WC, Weight Concern; PGA, Personal Goal Achievement; COM, Competition; REC, Recognition; AFF, Affiliation; PC, Psychological Coping; LM, Life Meaning; SE, Self-Esteem. Significant results are shown in bold (*p* < 0.05). Statistical tests: U, Mann–Whitney U test; H, Kruskal-Wallis test.

Income levels also played a significant role in shaping motivations. Participants with a net income above 3,000 PLN exhibited greater weight concern but scored lower in affiliation and self-esteem (*p* < 0.05). Financial difficulties were also shown to influence motivation negatively; individuals managing financial challenges scored significantly lower in personal goal achievement and competition (*p* < 0.05) compared to those with satisfactory material wellbeing. These findings emphasize the impact of financial security on both intrinsic and extrinsic motivational factors.

However, despite reaching statistical significance, the observed effect sizes were small, indicating that these socioeconomic factors explained only a limited proportion of the variance in motivational dimensions.

### Group differences in motivations across different socioeconomic characteristics

The significant main effects of selected socioeconomic factors on MOMS categories for each group of runners (RRs, MAs, and Us) are summarized in Table 5.

**Table 5:** Significant group differences in Motivations of Marathoners Scales (MOMS) across socioeconomic characteristics.

Main effects	Categories								
	HO	WC	PGA	COM	REC	AFF	PC	LM	SE
Education	Us					MAs, Us		MAs	MAs
Professional activity	RRs, MAs		RRs				MAs	MAs	MAs
Net income per capita		MAs				RRs, MAs			
Material wellbeing		Us	RRs	RRs					
Attitude toward material needs	Us	MAs, Us	MAs	MAs, Us	MAs, Us				

RRs, recreational runners; MAs, marathon runners; Us, ultramarathon runners; HO, Health Orientation; WC, Weight Concern; PGA, Personal Goal Achievement; COM, Competition; REC, Recognition; AFF, Affiliation; PC, Psychological Coping; LM, Life Meaning; SE, Self-Esteem.

#### Recreation runners

Professional activity: A significant effect was found for health orientation (*p* = 0.036, *η*^2^ = 0.02) as well as for personal goal achievement (PGA) (*p* = 0.002, *η*^2^ = 0.05). Although post-hoc analysis revealed no significant differences in health orientation, students exhibited significantly higher personal goal achievement than both self-employed individuals (*p* = 0.025) and retirees (*p* = 0.017). Regarding net income per capita, a significant effect on affiliation was observed (*p* = 0.018, *η*^2^ = 0.02), with individuals earning less than 3,000 PLN showing higher levels of affiliation. For marital wellbeing, significant effects were noted for personal goal achievement (*p* = 0.019, *η*^2^ = 0.03) and competition (*p* = 0.049, *η*^2^ = 0.02). However, *post-hoc* analysis did not indicate any significant differences. Overall, the effect sizes were small (*η*^2^ values ranging from 0.02 to 0.05), suggesting that while the associations are statistically significant, their practical impact may be limited.

#### Marathoners

Education demonstrated significant effects on affiliation (*p* < 0.001, *η*^2^ = 0.02), life meaning (*p* = 0.017, *η*^2^ = 0.01), and self-esteem (*p* = 0.008, *η*^2^ = 0.01). Notably, individuals with higher education reported lower levels in these areas than those with secondary education. Similarly, professional activity was significantly associated with health orientation (*p* = 0.045, *η*^2^ = 0.01), psychological coping (*p* = 0.008, *η*^2^ = 0.01), life meaning (*p* = 0.045, *η*^2^ = 0.01), and self-esteem (*p* = 0.021, *η*^2^ = 0.01). Although post-hoc analysis did not reveal significant differences in health orientation, students scored significantly higher in psychological coping (*p* = 0.004), life meaning (*p* = 0.036), and self-esteem (*p* = 0.014) than individuals in full-time employment. In the case of net income per capita, significant effects emerged for weight concern (*p* = 0.001, *η*^2^ = 0.01) and affiliation (*p* < 0.001, *η*^2^ = 0.02). Here, weight concern was more pronounced among individuals with higher incomes, while affiliation was stronger among those with lower incomes. Finally, attitude toward material needs was significantly related to weight concern (*p* < 0.001, *η*^2^ = 0.02), personal goal achievement (*p* = 0.004, *η*^2^ = 0.01), competition (*p* < 0.001, *η*^2^ = 0.02), and recognition (*p* = 0.002, *η*^2^ = 0.01). However, post-hoc analysis did not indicate any significant differences in these dimensions. Overall, like the RRs, the effect sizes in the MAs group were generally small (*η*^2^ ranging from 0.01 to 0.02), suggesting modest practical implications despite statistical significance.

#### Ultramarathoners

Education exhibited significant effects on health orientation (*p* = 0.01, *η*^2^ = 0.02) and affiliation (*p* = 0.04, *η*^2^ = 0.01), with individuals possessing higher education levels reporting lower scores in both domains. In addition, marital wellbeing was found to influence weight concern significantly (*p* = 0.038, *η*^2^ = 0.02), although post-hoc analysis did not reveal any significant differences. Furthermore, attitude toward material needs was significantly associated with multiple motivational types, including health orientation (*p* = 0.027, *η*^2^ = 0.01), weight concern (*p* < 0.001, *η*^2^ = 0.04), competition (*p* < 0.001, *η*^2^ = 0.04), and recognition (*p* = 0.002, *η*^2^ = 0.02). Individuals who placed a higher importance on material needs tended to score higher across these dimensions. While these results indicate several significant associations, the overall effect sizes remained relatively small.

### Relation between socioeconomic status and motivation in each group of runners

#### Education

No relationship was observed between socioeconomic status and motivation in the RRs group (*p* > 0.05). However, significant correlations were found between affiliation, life meaning, and self-esteem in the MAs group and health orientation and affiliation in the Us group. All correlations were notably negative, indicating that motivation increased with lower educational levels. The correlations were extremely weak despite the observed statistical significance (Table 6).

**Table 6:** The Spearman’s rank correlation coefficient between socioeconomic status (education level, net income per capita, material wellbeing, attitude toward material needs) and Motivations of Marathoners Scales (MOMS) in recreational runners (RRs), marathoners (MAs), and ultramarathon runners (Us).

Categories	Education			Net income per capita			Material wellbeing			Attitude toward material needs		
	RRs	MAs	Us	RRs	MAs	Us	RRs	MAs	Us	RRs	MAs	Us
Physical												
Health orientation	0.043	0.008	**−0.125**	−0.004	0.025	0.001	0.025	0.023	0.019	−0.095	0.033	**0.108**
Weight concern	0.071	−0.002	−0.055	0.077	**0.115**	0.083	0.018	0.030	**0.124**	0.098	**0.130**	**0.196**
Achievement												
Personal goal achievement	−0.047	−0.027	−0.040	0.022	0.026	0.040	0.073	0.051	0.026	0.073	**0.096**	0.081
Competition	−0.015	−0.044	0.000	−0.023	−0.016	0.025	0.102	0.027	0.086	0.051	**0.140**	**0.198**
Social												
Recognition	−0.018	−0.043	−0.055	0.001	−0.035	0.056	−0.014	−0.016	0.040	0.074	**0.109**	**0.149**
Affiliation	0.024	**−0.134**	**−0.100**	**−0.143**	**−0.126**	−0.042	−0.108	−0.015	0.025	−0.045	−0.032	0.012
Psychological												
Psychological coping	0.056	−0.013	−0.036	−0.066	−0.029	0.052	**−0.138**	−0.032	−0.053	0.005	0.011	0.002
Life meaning	−0.077	**−0.082**	−0.088	−0.087	−0.063	−0.007	−0.078	−0.006	−0.058	0.038	0.010	0.013
Self-esteem	−0.016	**−0.091**	−0.028	−0.082	−0.044	0.006	−0.073	−0.048	−0.043	0.072	0.062	0.074

RR, recreational runners; MA, marathon runners; U, ultramarathon runners; significant correlations are bolded (*p* < 0.05).

#### Net income per capita

No association was found between net income per capita and motivation in the Us group (*p* > 0.05). Meanwhile, significant negative correlations were observed between affiliation in the RRs and MAs groups, indicating a link between an increase in net income per capita and a decrease in this type of motivation. The net income per capita of the MAs group was associated with increased weight concerns. All correlations were extremely weak (Table 6).

#### Material wellbeing

There was no link between material wellbeing and motivation in the MAs group (*p* > 0.05). A significant negative correlation was observed in psychological coping within the RRs group, indicating that an increase in material wellbeing is associated with a decrease in psychological coping. Increased material wellbeing was associated with increased weight concerns in the Us group. All the correlations were characterized as very weak (Table 6).

#### Attitude toward material needs

No relationship was found between socioeconomic status and motivation in the RRs group (*p* > 0.05). However, significant positive correlations were observed for weight concerns, competition, and recognition in the RRs and MAs groups, indicating that the higher the importance placed on material needs, the higher the motivation associated with these three categories. Significant positive correlations were also found in the Us group regarding health orientation and in the MAs group regarding personal goal achievement. All correlations were considered extremely weak (Table 6).

### Differences in the number of completed marathons and ultramarathons across socioeconomic characteristics

Participation in marathons and ultramarathons varied significantly across different socioeconomic groups (Table 7). Professional activity emerged as a significant determinant of marathon participation (*p* < 0.05), with full-time employees and self-employed individuals completing more marathons than students or unemployed participants. This result suggests that occupational stability facilitates long-term commitment to endurance sports. Similarly, net income per capita was significantly associated with marathon participation, as higher-income individuals completed more marathons than those with lower incomes (*p* < 0.05).

**Table 7:** Differences in the number of completed marathons and ultramarathons across socioeconomic characteristics.

Variables	Number of marathons finished						Number of ultramarathons finished				
	0	1–3	4–10	10–30	More than 30	*p* (*ω*)	0	1–3	4–10	More than 10	*p* (*ω*)
Education, *n* (%)											
Higher	206 (69.59%)	478 (73.99%)	291 (72.93%)	108 (73.97%)	38 (73.08%)	0.716 (0.04)	806 (72.22%)	175 (72.02%)	103 (81.10%)	37 (69.81%)	0.177 (0.06)
Secondary	90 (30.41%)	168 (26.01%)	108 (27.07%)	38 (26.03%)	14 (26.92%)		310 (27.78%)	68 (27.98%)	24 (18.90%)	16 (30.19%)	
Professional activity, *n* (%)											
Full-time employment	215 (72.64%)	506 (78.33%)	311 (77.94%)	116 (79.45%)	38 (73.08%)	**<0.001** (0.25)	860 (77.06%)	187 (76.95%)	101 (79.53%)	38 (71.70%)	**0.002** (0.13)
Self-employment	21 (7.09%)	78 (12.07%)	49 (12.28%)	20 (13.70%)	6 (11.54%)		116 (10.39%)	31 (12.76%)	17 (13.39%)	10 (18.87%)	
Student	53 (17.91%)	55 (8.51%)	19 (4.76%)	3 (2.05%)	0 (0.00%)		111 (9.95%)	14 (5.76%)	5 (3.94%)	0 (0.00%)	
Unemployed	7 (2.36%)	7 (1.08%)	20 (5.01%)	7 (4.79%)	8 (15.38%)		29 (2.60%)	11 (4.53%)	4 (3.15%)	5 (9.43%)	
Net income per capita, *n* (%)											
>3 k	89 (30.07%)	247 (38.24%)	135 (33.83%)	67 (45.89%)	15 (28.85%)	**0.007** (0.1)	397 (35.57%)	86 (35.39%)	49 (38.58%)	21 (39.62%)	0.851 (0.02)
≤3 k	207 (69.93%)	399 (61.76%)	264 (66.17%)	79 (54.11%)	37 (71.15%)		719 (64.43%)	157 (64.61%)	78 (61.42%)	32 (60.38%)	
Material wellbeing, *n* (%)											
I can afford everything I dream of	8 (2.70%)	21 (3.25%)	11 (2.76%)	11 (7.53%)	2 (3.85%)	0.328 (0.1)	40 (3.58%)	5 (2.06%)	4 (3.15%)	4 (7.55%)	0.331 (0.08)
I satisfy my needs to a satisfactory degree	224 (75.68%)	506 (78.33%)	306 (76.69%)	114 (78.08%)	41 (78.85%)		853 (76.43%)	197 (81.07%)	100 (78.74%)	41 (77.36%)	
I satisfy my needs to the minimum extent	23 (7.77%)	35 (5.42%)	29 (7.27%)	8 (5.48%)	2 (3.85%)		78 (6.99%)	13 (5.35%)	6 (4.72%)	0 (0.00%)	
I am dealing, but I often have financial problems	41 (13.85%)	84 (13.00%)	53 (13.28%)	13 (8.90%)	7 (13.46%)		145 (12.99%)	28 (11.52%)	17 (13.39%)	8 (15.09%)	
Attitude toward material needs, *n* (%)											
Important to me	145 (48.99%)	316 (48.92%)	181 (45.36%)	76 (52.05%)	22 (42.31%)	0.545 (0.04)	554 (49.64%)	116 (47.74%)	51 (40.16%)	19 (35.85%)	0.056 (0.07)
Irrelevant to me	151 (51.01%)	330 (51.08%)	218 (54.64%)	70 (47.95%)	30 (57.69%)		562 (50.36%)	127 (52.26%)	76 (59.84%)	34 (64.15%)	

Significant results are shown in bold (*p* < 0.05).

The analysis of ultramarathon participation revealed fewer significant socioeconomic associations, although professional activity remained an influential factor. Full-time employees demonstrated higher levels of ultramarathon participation than other groups, reflecting their greater capacity to manage such events’ financial and time-related demands. Interestingly, students’ participation in marathons and ultramarathons declined as the number of completed events increased, indicating that their involvement may be more sporadic or exploratory.

## Discussion

This study examined how SES influences participation patterns and motivation in endurance sports, particularly among RRs, MAs, and Us. The findings confirm that higher-SES individuals are more likely to participate in structured endurance events due to financial and professional stability. At the same time, ultramarathons attract participants driven more by intrinsic motivation, resilience, and self-determination rather than financial resources.

Motivational differences across groups followed expected trends, with ultramarathoners prioritizing goal achievement and self-esteem, while recreational runners emphasized affiliation. However, SES also shaped these motivations, with higher-SES individuals focusing on achievement and self-esteem, while lower-SES participants prioritized health and social engagement. Financial stability facilitated participation and shifted motivation toward extrinsic factors like weight concern, whereas financial constraints suppressed competitiveness and goal-driven behaviors.

While our results indicate statistically significant differences in motivation across SES groups, it is essential to acknowledge that the effect sizes were generally small. This means that while a relationship exists, its practical impact is limited, suggesting that SES is only one of many factors influencing motivation in endurance sports. This indicates that although SES plays a role in shaping motivation, other factors such as personality traits, cultural norms, and individual life experiences may exert stronger influences (Breuer et al., 2010). The small effect sizes align with findings in sports psychology, where multiple overlapping determinants contribute to motivation (Eime et al., 2010). Thus, while our results provide valuable insights, they should be interpreted within the broader framework of endurance sports participation, where intrinsic and extrinsic motivators interact in complex ways.

The negligible effect size observed for the relationship between SES and goal achievement motivation suggests that while financial stability and education may facilitate participation, they are not the sole determinants of an athlete’s drive for achievement. Instead, intrinsic psychological factors like self-efficacy and resilience likely dominate ultramarathon participation (Corrion et al., 2018). Similarly, the influence of SES on self-esteem motivation, though statistically significant, remains small, indicating that endurance sports may serve as an equalizer where personal determination outweighs socioeconomic constraints.

This discussion explores how education, income, and professional activity shape endurance sports participation and motivation while addressing strategies to promote accessibility and long-term engagement across socioeconomic groups.

### Socioeconomic characteristics of RRs, Mas, and Us

Our hypothesis proposed that SES significantly influences participation patterns in endurance sports, with individuals of higher SES more likely to participate in long-distance events such as marathons and ultramarathons. The findings support this assumption, particularly regarding professional activity, which strongly impacted participation in both events. Full-time employment and self-employment offered the financial stability and structured time management needed for sustained engagement in endurance sports. In contrast, students participated less frequently, likely due to their transient life circumstances, economic constraints, and competing academic demands.

These results align with Guddal et al. (2017), who found that SES-related disparities in sports participation often emerge from financial and social constraints rather than intrinsic motivation differences. Similarly, Breuer et al. (2010) emphasized that higher-SES individuals have greater access to structured training, specialized coaching, and travel opportunities, making endurance sports more feasible. This advantage is evident in our findings, as participants with full-time employment and stable careers were more likely to engage in long-distance running events. Scheerder et al. (2015b) further noted that lower-SES individuals face more barriers due to financial constraints and a lack of flexible work schedules, a trend that appears to hold in our study as well.

A particularly important factor was income level, which played a significant role in marathon participation and reinforced financial barriers that may limit accessibility for lower-income individuals. Despite the growing popularity of endurance sports, economic constraints remain a significant challenge, especially for organized events that necessitate travel, gear, and entry fees. Our results indicated that higher-income individuals participated more frequently in organized marathons, demonstrating that financial barriers can hinder lower-income groups from accessing these events. Thuany et al. (2021) observed similar findings among Brazilian runners, where higher socioeconomic status correlated with increased participation in structured races. These findings align with those of Hallmann et al. (2011), who reported that the cost of race participation can discourage lower-income individuals, even if they have the motivation to compete. Similarly, Krouse et al. (2011) highlighted that lower-income women face financial challenges in endurance sports. Eime et al. (2010) noted that lower-SES runners tend to rely on community-based events rather than commercial races, reinforcing the pattern seen in our results.

Interestingly, while income strongly correlated with marathon participation, it showed a weaker correlation with ultramarathon participation (Table 6). This suggests that ultramarathons attract a unique subset of participants motivated more by personal challenge than financial feasibility. Our results showed that ultramarathoners displayed high motivation despite economic constraints, suggesting that other psychological or social factors may outweigh financial limitations. Corrion et al. (2018) support this, finding that ultramarathoners are often driven by mental resilience and self-exploration rather than competitive or economic incentives.

Gerasimuk et al. (2021) also emphasized that ultramarathon participants exhibit strong motivation for goal achievement, which can override financial considerations. This corresponds to our findings, where participants in ultra-distance events reported a strong commitment to personal growth and goal setting. Additionally, Kodli (2016) suggested that while SES influences the type of sports individuals engage in, ultramarathons are less influenced by wealth and more by internal psychological motivations. These results are further supported by Mayolas-Pi et al. (2017), who argued that financial constraints do not significantly deter ultramarathon participation. Still, they may impact long-term retention due to the cumulative costs of gear, nutrition, and travel. Krouse et al. (2011) found that while entry into ultramarathons is not necessarily hindered by SES, maintaining participation over time requires financial flexibility for training and event costs. Coe et al. (2013) suggest that successful endurance fitness often develops cost-effective strategies to continue participating, which may explain why SES played a lesser role in their event choice.

Socioeconomic status, particularly professional engagement, was a key factor among groups involved in endurance sports. Our findings confirmed that full-time employment was the dominant category across all groups, highlighting the significance of occupational stability (Table 2). However, variations in secondary daily activities suggest that lifestyle and time-management choices were adjusted to meet the demands and commitments of endurance training. These results align with Partyka and Waśkiewicz (2024), who noted that athletes with stable employment structures are more likely to sustain long-term participation in endurance sports. Interestingly, education, net income per capita, material wellbeing, and attitudes toward material needs were evenly distributed among the groups, indicating that beyond a certain threshold, these factors cease to influence one’s inclination toward endurance sports significantly. This trend echoes the findings of Grzywacz and Marks (2001), who discovered that while SES affects physical activity levels, the effects plateau beyond a specific financial threshold. Similarly, Studer et al. (2011) argued that although wealthier individuals may have better access to sports facilities, motivation and lifestyle factors become critical determinants of long-term involvement. The broader implications of these findings are also reflected in the work of Duetz et al. (2003), who explored how socioeconomic status and cultural context influence fitness and health outcomes. They found that in affluent societies such as Switzerland, higher SES is positively associated with improved fitness and health, while lower SES correlates with higher morbidity and mortality rates. These findings resonate with our results, as higher-SES individuals in our study reported more consistent participation in endurance sports, likely contributing to overall better health and wellbeing. Meyer et al. (2005) further supported these claims in their study of 8,405 Swiss citizens aged 50–80, demonstrating that higher SES is linked to greater engagement in moderate-to-vigorous sports activities. Conversely, lower-SES individuals were more likely to engage in inconsistent or chronic physical inactivity, reinforcing our findings that financial and occupational stability promote engagement in endurance sports.

### Differences in motivation across RRs, MAs, Us

Our hypothesis suggested that socioeconomic factors would influence motivation in endurance sports, with higher-SES individuals exhibiting more potent intrinsic motivators like goal achievement and self-esteem. The motivational aspect of this study clarified the psychological underpinnings of endurance sports participation. Most motivation scores were high, reflecting participants’ robust intrinsic drive. Personal goal achievement was the dominant motivation across all groups, especially among ultramarathoners, suggesting that challenging endurance sports are strongly linked to personal milestones. Self-esteem and weight concerns were also significant motivational factors, particularly among ultramarathoners, indicating possible self-affirmation and health consciousness associated with such demanding physical activities. The findings suggest that education is crucial in shaping interpersonal and self-worth-related motivations such as affiliation and self-esteem. This aligns with previous research suggesting that higher education enhances social and psychological resources, promoting involvement in endurance sports (Maklári et al., 2024; Tóth et al., 2024). Similarly, Partyka and Waśkiewicz (2024) found that higher-educated marathon runners are more likely to participate in endurance sports for self-fulfillment rather than external rewards, reinforcing that intrinsic motivation is connected to intellectual engagement. The significantly higher scores in personal goal achievement among students indicate that at this stage of life, individual aspirations and self-improvement are primary motivators (Eime et al., 2010; Hallmann et al., 2011). Income further revealed a nuanced relationship with motivation. While financial stability facilitates participation, it may shift motivations toward extrinsic factors, such as Weight Concern, suggesting that higher-income individuals may prioritize esthetic or performance-related outcomes over social or intrinsic benefits (Chylińska, 2024; Krouse et al., 2011; Mayolas-Pi et al., 2017; Wilczynska et al., 2021). Conversely, financial stress appeared to suppress ambition and competitiveness, as seen in lower scores for Personal Goal Achievement and Competition motivation among lower-income runners. These findings support Singh and Boruah (2024), who observed that financial insecurity limits access to structured training environments, thereby hindering an athlete’s ability to develop a long-term commitment to endurance sports (Breuer et al., 2010; Krouse et al., 2011; Vandendriessche et al., 2012).

Overall, the second hypothesis was only partially supported. Ultramarathoners showed higher goal achievement and self-esteem motivation than recreational runners, but affiliation motivation was higher in recreational runners, and no significant differences emerged for life-meaning motivation. While personal goal achievement and social affiliation showed clear trends in line with the hypothesis, health orientation and self-esteem motivations showed mixed evidence (Blum, 2024; Gerasimuk et al., 2021). The Kruskal-Wallis test confirmed significant motivational differences across groups, yet small eta-squared (*η*^2^) values (0.00–0.01) suggest negligible effect sizes. This may mean that the grouping variable explains only slight variance, indicating that other factors—such as individual traits, experiences, and socioeconomic context—could play a more significant role in shaping motivation (Eren, 2017; Thuany et al., 2021; Scheerder et al., 2015b).

Since the original data were gathered, marathon and ultramarathon running has expanded globally, diversified in age and gender participation, and become more accessible through virtual events and community-based initiatives. Technological innovations—such as wearable fitness trackers and online training platforms—now offer runners enhanced goal-setting and progress-monitoring tools (Migliaccio et al., 2024; Schumann and Doherty, 2024). At the same time, cultural narratives increasingly emphasize the mental health and community-building benefits of endurance sports (Beasley and Johnson-Pack, 2024; Busanich et al., 2012).

Despite these contextual shifts, no recent literature indicates that the fundamental motivational drivers (e.g., achievement, self-esteem, social affiliation, and health orientation) have changed significantly. Although the modalities and environments for engaging in long-distance running have evolved, no significant evidence suggests a transformation in participation’s underlying psychological or socioeconomic determinants (Fogaça et al., 2024; Partyka and Waśkiewicz, 2024). Accordingly, the insights presented in this study continue to be relevant to understanding the interplay between socioeconomic status and runner motivation. However, the abovementioned developments reinforce the need for ongoing research to validate these findings and explore how technological and cultural changes might further refine or augment motivation in endurance sports.

### SES and motivation across RRs, MAs, and Us

#### Education and motivation: the role of cognitive and social capital

Education emerged as a crucial factor shaping affiliation, self-esteem, and life-meaning motivation, with higher-educated individuals exhibiting significantly higher scores in these areas (Table 4). These results align with previous research showing that education enhances social and psychological resources, which may translate into a stronger inclination for self-improvement and social engagement in endurance sports (Maklári et al., 2024; Sánchez-García et al., 2024). Higher education also fosters long-term goal orientation and cognitive discipline, traits that are often associated with intrinsic forms of motivation such as personal growth, resilience, and self-actualization (Tóth et al., 2024). The negative association between education and affiliation motivation among ultramarathoners (Table 5) suggests that at higher levels of endurance sport engagement, motivations may shift from social connection toward individual perseverance and psychological endurance. This aligns with findings from Partyka and Waśkiewicz (2024), who reported that higher-educated runners often prioritize self-fulfillment and goal achievement over social or recognition-based motives.

Moreover, students’ notably higher scores in personal goal achievement support the idea that early adulthood is a period of strong intrinsic drive for self-improvement (SHI Shi et al., 2024). These findings corroborate those of Eime et al. (2010) and Hallmann et al. (2011) who observed that younger adults often engage in endurance sports as a means of self-discovery, identity formation, and competence building.

#### Income and the shift between intrinsic and extrinsic motivations

The findings suggest that financial stability shifts motivation from intrinsic to extrinsic factors, with higher-income participants exhibiting more significant weight concern but lower affiliation and self-esteem motivation (Table 4). These results support previous research indicating that higher-income individuals are more likely to frame endurance sports to achieve performance or esthetic goals rather than for social connection or self-worth enhancement (Chylińska, 2024; Krouse et al., 2011).

The inverse relationship between income and affiliation motivation suggests that social interaction may be a key driver of endurance sport participation among lower-income individuals, who might lack alternative avenues for structured social engagement (Mayolas-Pi et al., 2017; Wilczynska et al., 2021). This trend was particularly evident among marathoners, where lower-income individuals reported stronger affiliation motivation, while higher-income runners placed greater emphasis on body image and weight management (Table 5).

Conversely, financial stress appeared to suppress ambition and competitiveness, as seen in the lower scores for personal goal achievement and competition motivation among lower-income runners (Table 4). This aligns with Breuer et al.’s (2011) and Singh and Boruah’s (2024) findings, highlighting how economic insecurity limits access to structured training, high-quality coaching, and competitive opportunities, ultimately reducing long-term athletic engagement.

These results also resonate with Vandendriessche et al. (2012), who found that lower-income athletes often prioritize endurance sports for physical health and psychological wellbeing, while wealthier participants are more likely to focus on measurable performance improvements. Thuany et al. (2021) observed similar patterns in Brazilian endurance athletes, reporting that economic resources significantly shape participation intensity and motivational structure.

#### Professional activity and motivation: the work-sport balance

Occupational status played a key role in shaping motivation, with students demonstrating the highest personal goal achievement scores, reinforcing that this life stage prioritizes aspirations for self-improvement (Table 5) (Eime et al., 2010; Hallmann et al., 2011). The data also showed that marathoners and ultramarathoners in full-time employment exhibited stronger psychological coping motivation, reflecting the well-documented association between sports participation and stress management among working professionals (Ávalos-Ramos et al., 2024).

Interestingly, unemployed individuals reported lower personal goal achievement and competition motivation, reinforcing that economic instability and career uncertainty may suppress long-term ambition in endurance sports. This supports the work of Partyka and Waśkiewicz (2024), who found that stable employment structures facilitate sustained engagement in endurance sports by providing financial and psychological security.

The relationship between self-employment and endurance sports participation is also noteworthy. Self-employed individuals exhibited moderate levels of personal goal achievement motivation, suggesting that flexible work schedules may enable consistent training but do not necessarily heighten competitive ambition. This aligns with previous findings that entrepreneurs and freelancers often use endurance sports as a self-regulatory mechanism rather than a performance-driven pursuit (Scheerder et al., 2015a).

#### Material wellbeing and attitudes toward material needs

Self-perceived financial wellbeing was a significant predictor of personal goal achievement and competition motivation, with financially secure individuals scoring higher in both categories (Table 4). These results align with Breuer et al.'s (2010) and Funk and Bruun (2007) findings, who noted that financial security facilitates goal-oriented motivation in endurance sports by reducing external pressures and allowing for greater focus on structured training and performance outcomes. However, a greater emphasis on material needs was associated with increased weight concern, competition, and recognition motivation (Table 5). This suggests that individuals prioritizing financial success may be more inclined toward performance-oriented running, where external validation and body image considerations play a more significant role (Thuany et al., 2021). Interestingly, material wellbeing had no significant impact on health orientation or life-meaning motivation, reinforcing the notion that these forms of motivation are more stable across SES levels and do not fluctuate based on financial status (Mulderij et al., 2022; Śmigielski et al., 2024). This suggests that runners across all economic backgrounds recognize endurance sports as a pathway to mental and physical wellbeing, independent of financial standing.

The association between material wellbeing and competition motivation was robust among ultramarathoners, indicating that financial stability may promote a more competitive and performance-driven approach to extreme endurance events (Table 5). This aligns with previous research by Blum (2024) and Myburgh and Kruger (2024), who found that ultramarathoners with financial resources tend to focus on achieving prestigious race completions and improving performance metrics rather than participation for health or recreation.

### Socioeconomic determinants of participation patterns

Participation in endurance events also reflected socioeconomic disparities. The significant influence of professional activity on both marathon and ultramarathon participation underscores the importance of occupational stability in facilitating long-term commitment to these demanding sports. Full-time employment and self-employment likely provide the financial resources and structured time management necessary for consistent participation (Lorin Braschler et al., 2024; Maklári et al., 2024). In contrast, the sporadic involvement of students may reflect their transient life circumstances and competing priorities, limiting their ability to train and participate in endurance consistently.

The role of income in marathon participation reinforces the financial barriers that may limit access for lower-income individuals, despite the growing popularity of endurance sports (Breuer et al., 2010; Corrion et al., 2018; Wicker et al., 2012). However, the weaker association between income and ultramarathon participation suggests that these events may attract a subset of participants whose motivations transcend financial constraints. This finding warrants further exploration to identify the unique characteristics of ultramarathon participants, including potential differences in psychological resilience and self-determination (Blum, 2024; Myburgh and Kruger, 2024).

These findings emphasize the need for targeted interventions to improve accessibility and engagement across socioeconomic backgrounds. Addressing financial barriers through sponsorships, reduced entry fees, or community-based funding programs could help level the playing field for lower-income participants (Hirvensalo and Lintunen, 2011). Furthermore, leveraging educational resources and fostering intrinsic motivations such as Affiliation and Life Meaning may enhance participation across diverse groups, especially among students and early-career professionals. Recognizing the unique motivations of different demographic groups—students, professionals, and lower-income athletes—can inform strategic outreach efforts to ensure the long-term sustainability of endurance sports participation (Freitas et al., 2007; Irandoust, 2020).

### Limitations and future directions

This study has several limitations. The sample’s cultural, geographic, or socioeconomic background may limit generalizability. A post hoc power analysis revealed that the study was highly powered, suggesting that the large sample size led to the detection of minimal effects, though effect sizes were minimal. This indicates that while statistically significant, the practical significance of these effects is limited. Future studies could refine the sample size or explore alternative variables that better capture motivational differences.

Cross-sectional data restrict causal inferences, and self-reported measures may introduce bias. Additionally, the data collection period (January to March 2008) may not fully reflect recent trends in marathon and ultramarathon running. Although age and gender were not specifically analyzed in this study, future research should consider these variables to better understand their potential influence on motivations in different runner groups.

Future research should incorporate diverse cultural and geographic contexts, combine objective and self-reported measures, and consider longitudinal designs to explore causal relationships and evolving motivations. Integrating interdisciplinary approaches and updated data will provide a more comprehensive understanding of contemporary runner motivations and the role of SES in endurance sports.

## Conclusion

This study highlights the role of SES in endurance sports participation. Higher-SES individuals were more likely to take part in marathons and ultramarathons, likely due to financial and professional stability, though ultramarathon participation appeared to depend more on intrinsic motivation than financial resources.

Motivational priorities differed across groups: Us emphasized goal achievement and self-esteem, while recreational runners prioritized social affiliation. MSs showed a balance of both. However, small effect sizes suggest that personality, experience, and socioeconomic context may be stronger influences than event classification alone. SES also shaped motivational drivers—higher-SES individuals focused on achievement and self-esteem, whereas lower-SES runners were more motivated by health benefits and social affiliation. However, weak correlations indicate that broader psychological, social, and cultural factors also play a role.

Finally, higher SES correlated with more completed marathons and ultramarathons, likely due to financial resources and better training access. However, the weaker link for ultramarathons suggests that intrinsic motivation may help overcome financial constraints. Sponsorship and community programs could support lower-SES athletes in maintaining long-term participation.

These findings should be interpreted with consideration, as the small effect sizes suggest that the influence of SES on motivation in endurance sports is present but limited in magnitude.

## Data Availability

The raw data supporting the conclusions of this article will be made available by the authors without undue reservation.
